# Identification of Vascular and Hematopoietic Genes Downstream of *etsrp* by Deep Sequencing in Zebrafish

**DOI:** 10.1371/journal.pone.0031658

**Published:** 2012-03-16

**Authors:** Gustavo Gomez, Jae-Hyung Lee, Matthew B. Veldman, Jing Lu, Xinshu Xiao, Shuo Lin

**Affiliations:** 1 Department of Molecular, Cell and Developmental Biology, University of California Los Angeles, Los Angeles, California, United States of America; 2 Department of Integrative Biology and Physiology, University of California Los Angeles, Los Angeles, California, United States of America; University of Sheffield, United Kingdom

## Abstract

The transcription factor *etsrp*/*Er71*/*Etv2* is a master control gene for vasculogenesis in all species studied to date. It is also required for hematopoiesis in zebrafish and mice. Several novel genes expressed in vasculature have been identified through transcriptional profiling of zebrafish embryos overexpressing *etsrp* by microarrays. Here we re-examined this transcriptional profile by Illumina RNA-sequencing technology, revealing a substantially increased number of candidate genes regulated by *etsrp*. Expression studies of 50 selected candidate genes from this dataset resulted in the identification of 39 new genes that are expressed in vascular cells. Regulation of these genes by *etsrp* was confirmed by their ectopic induction in *etsrp* overexpressing and decreased expression in *etsrp* deficient embryos. Our studies demonstrate the effectiveness of the RNA-sequencing technology to identify biologically relevant genes in zebrfish and produced a comprehensive profile of genes previously unexplored in vascular endothelial cell biology.

## Introduction

The cardiovasacular system, which includes the heart, vessels and blood, function together to deliver oxygen and nutrients to cells throughout the body and remove metabolic waste. Understanding the development of this system is instrumental to the advancement of both basic and clinical sciences. The zebrafish, *Danio rerio*, is an excellent model organism for such studies due to embryo transparency, high fecundity, and fast development of organogenesis. In particular, the cardiovascular system is formed within one day of birth [1], [2]. Through genetic, cellular and molecular studies in zebrafish, a great deal of knowledge regarding the molecular components and cellular events that establish this system has been obtained. It is notable that many of the key molecular players and events that drive organogenesis in zebrafish are evolutionarily and functionally conserved with other organisms, including mammals.

A critical transcription factor required for the development of vascular endothelial cells is *ets related protein*, *etsrp*, which was originally discovered through transcriptional profiling of the cardiovascular mutant line, *cloche*, that lacks blood and vasculature [3] and independently by a mutagenesis screen [4]. It was subsequently found that *etsrp* is both necessary and sufficient to induce both the vascular endothelial and primitive myelopoietic program in zebrafish [5], [6]. Its significance was further underscored by the identification and characterization of the functional homolog *ets variant 2*, *etv2*, in mammals [7], [8] and Xenopus [9], [10]. The *etsrp* transgenic fish line has been useful to reveal the cellular events that establish the cranial vasculature and myelopoiesis [11], and it is possible that *etsrp* might be associated with the initiation of the definitive hematopoietic program [12].

The zebrafish genome is now in its ninth version and remains incompletely annotated. Nonetheless bioinformatic approaches have been used to identify important genes encoding transcription factors containing the ETS box DNA binding domain in hematopoietic and endothelial development [13]. The use of microarrays by the zebrafish research community has increased the identification of genes expressed in the developing cardiovascular system [3], [14], [15], [16], [17], [18], [19]. The arrival of higher throughput next generation sequencing has expanded the possibilities to deepen our understanding and identification of novel genes, and has already proven its utility for studies in zebrafish. Although the majority of the published studies combining RNA-sequencing and zebrafish have focused on immunity [20], [21], [22], the use of high throughput sequencing to study other biological subjects in zebrafish has begun to increase [23], [24], [25]. The ability of this technological approach to examine the zebrafish transcriptome at greater depth than microarrays without an *a priori* bias prompted us to re-examine the transcriptional profile of embryos overexpressing *etsrp*. In this study we present a panel of 39 more genes that are expressed in the developing zebrafish vasculature that were identified by this approach.

## Results

**Figure 1:**
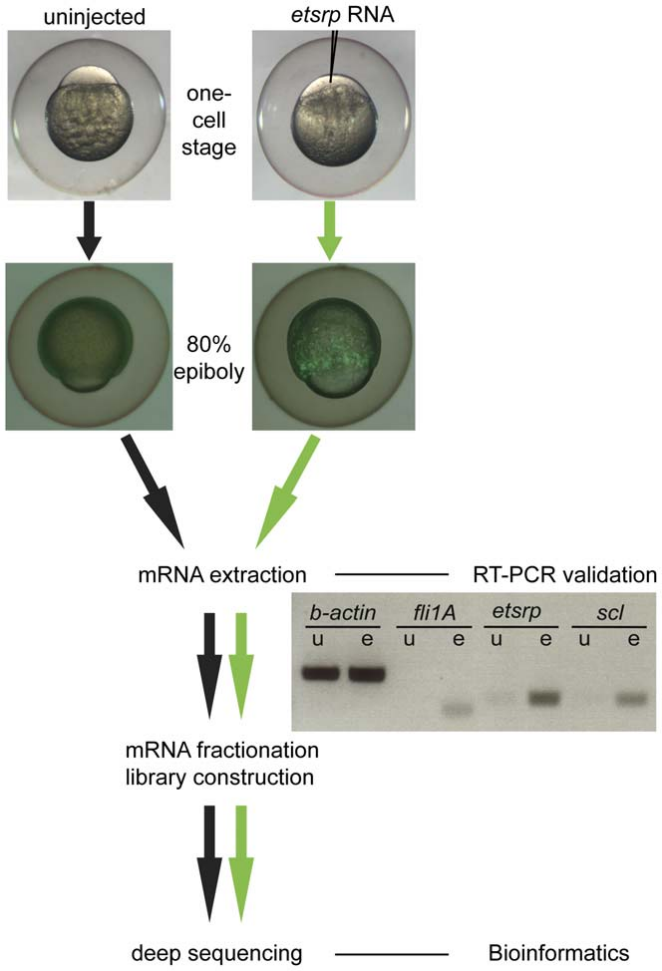
Workflow diagram. Embryos were either uninjected or injected with *etsrp* RNA at the one cell stage, then raised until the late gastrulation stages when the *flk1-gfp* transgenic reporter is induced ectopically. Equal paired groups of embryos were pooled for mRNA extraction, library construction, and solexa mRNA-sequencing. Samples were pre-validated prior to sequencing by RT-PCR with *fli1A*, the 3′UTR of *etsrp*, as well as *scl*, which were all preferentially induced as expected by *etsrp* overexpression (e) relative to uninjected controls (u).

As previously demonstrated, the overexpression of *etsrp* results in the induction of vascular related genes during gastrulation stages of development, before the onset of angioblast specification [5]. In this study *etsrp* RNA was injected into one cell *flk1-gfp* transgenic embryos and their transcriptome profiles were examined at later stages of gastrulation, 70–90% epiboly, when ectopic induction of *flk1-gfp* is detected (Figure 1). Total RNA was extracted from pools of 125 embryos, and the induction of known hemangioblast markers, *fli1a*, *scl* and *etsrp*, was verified in the *etsrp* overexpressed group by RT-PCR before library construction and sequencing. Two separate sets of RNA-seq libraries were constructed from independent samples that were pre-validated using the same validation scheme as demonstrated in the workflow diagram on Figure 1.

A total of 123 million single-end or paired-end reads were obtained and 35% of the reads were mapped onto the unigene transcript sequence database (Build #117) [26] using the Burrows Wheeler Aligner (BWA) alignment program [27]. Table S1 lists the number of reads in each control and *estrp* overexpressed (*estrp* oe) samples and the mapping results. About 43 million reads were uniquely mapped in total, which covered 77% of the unigene database (39,784 out of 51,481). The expression level difference between the control and *estrp* oe samples were compared, resulting in the upregulation of 849 unigene entries above a 1.9-fold cutoff value in the *etsrp* oe group with a corrected p-value<0.05, while 726 entries were downregulated using the same parameters. A sample of the top 35 hits of annotated upregulated genes obtained in this dataset is listed in Table 1.

**Table 1:** Top 35 annotated genes induced by *etsrp*.

Unigene ID	Gene ID	Gene Description	Fold Change	p-value
Dr.24158	*srgn*	*serglycin*	1920.8	7.7E-53
Dr.153782	*LOC100151241*	*similar to loc559821 protein*	1899.9	4.5E-26
Dr.83594	*crabp1a*	*cellular retinoic acid binding protein 1a*	917.4	5.1E-17
Dr.141228	*LOC558783*	*similar to cell death activator cide-a*	452.8	1.1E-05
Dr.149118	*LOC798186*	*similar to cdc42 gtpase-activating protein*	441.5	1.9E-21
Dr.118570	*LOC561493*	*similar to src homology 2 domain containing e*	315.3	1.8E-196
Dr.40434	*ms4a17a.11*	*membrane-spanning 4-domains, subfamily a, member 17a.11*	300.4	3.5E-06
Dr.133475	*LOC100151130*	*similar to rasip1 protein*	254.8	3.6E-10
Dr.37870	*ckmb*	*creatine kinase, muscle b*	231.6	3.8E-07
Dr.92232	*efcab2*	*ef-hand calcium binding domain 2*	231.5	8.4E-03
Dr.84343	*zgc:153721*	*zgc:153721*	225.9	1.1E-09
Dr.85502	*mrc1*	*mannose receptor c1-like protein*	225.7	4.0E-20
Dr.47591	*etv2*	*ets variant gene 2*	219.0	4.9E-237
Dr.110840	*LOC100006361*	*similar to preprogalanin 1b*	217.2	4.9E-03
Dr.103328	*zgc:162298*	*zgc:162298*	198.7	2.0E-05
Dr.86665	*nhlh2*	*nescient helix loop helix 2*	180.6	2.1E-08
Dr.114377	*zgc:173594*	*zgc:173594*	175.7	2.8E-03
Dr.110713	*LOC100149611*	*similar to loc495463 protein*	165.4	3.8E-08
Dr.84866	*LOC100005159*	*similar to hemicentin 1*	159.4	9.6E-04
Dr.111220	*gpr183*	*g protein-coupled receptor 183*	151.4	1.9E-04
Dr.113631	*LOC568486*	*similar to regulating synaptic membrane exocytosis 1*	136.1	2.5E-02
Dr.82184	*LOC569386*	*platelet endothelial cell adhesion molecule-like*	123.4	6.2E-06
Dr.124732	*LOC100148619*	*similar to myelin associated glycoprotein*	113.9	1.4E-02
Dr.152900	*LOC572378*	*similar to transforming growth factor-b type ii receptor*	110.1	4.9E-03
Dr.142587	*csf3r*	*colony stimulating factor 3 receptor (granulocyte)*	104.9	6.5E-05
Dr.100658	*LOC100004843*	*hypothetical loc100004843*	104.1	2.8E-03
Dr.149641	*LOC558126*	*similar to hcg2040171*	103.1	4.9E-03
Dr.151919	*si:ch211-10e8.4*	*si:ch211-10e8.4*	102.9	8.4E-03
Dr.81739	*kcnh2*	*potassium voltage-gated channel, subfamily h, member 2*	101.5	2.0E-09
Dr.78041	*xirp2*	*xin actin-binding repeat containing 2*	95.0	7.6E-56
Dr.118849	*zgc:63958*	*zgc:63958*	91.4	1.7E-03
Dr.102595	*LOC568153*	*similar to chemokine receptor-like 1*	86.4	2.8E-03
Dr.115871	*LOC569038*	*similar to cholecystokinin a receptor*	85.0	2.5E-02
Dr.83127	*LOC100001838*	*similar to loc100001772 protein*	79.5	1.4E-02

Several unigene hits identified in the top list correspond to unannotated expressed sequence tags, est's, and these were removed from this table. The dataset containing the data in its entirety including the est entries that were removed from this table are on Table S7.

In order to determine the biological relevance of the gene pool obtained here, a gene ontology analysis was performed with the Database for Annotation, Visualization and Integrated Discovery Gene Ontology (DAVID GO) program, as demonstrated in the identification of novel genes expressed in myeloid cells in zebrafish [28]. Of the 849 upregulated unigene hits examined by functional annotation clustering, 797 were queried successfully, but because some unigene entries are associated with more than one gene, and some genes are represented by more than one unigene identifier, a total of 787 entries were evaluated resulting in 148 clusters. The remaining 52 entries were not identified either because they represent expressed sequence tags not associated with a characterized gene, they are unique to zebrafish, or their orthologs were unidentifiable by DAVID GO gene conversion tools. Conversely, the 726 downregulated hits resulted in the successful query of 306 entries divided among 50 clusters, but relative to the induced clusters, these had rather low fold-enrichment scores.

The top two clusters from the upregulated data contained genes associated with “vascular development”, “angiogenesis,” and other terms related to vascular endothelial cell biology. These data were combined resulting in 68 genes after duplicate deletion, and examined individually for involvement in vascular development. 18 genes were excluded because although several are expressed in mesoderm or heart, their specific expression or function within endothelial cells remains to be demonstrated. We did examine one of these, *xirp2a*, which we found to have endothelial expression. These were noted as a subset of potential vascular genes in Table S2, which also includes 12 that were removed because there is no indication that they are associated with endothelial cells. By searching the list generated containing 849 unigene entries, 15 more vascular related genes were identified (Table 2), bringing the total number of genes associated with endothelial cells in this dataset to 53.

**Table 2:** Known vascular genes induced by *etsrp* in RNA-seq dataset.

Unigene ID	Gene ID	Gene Description	Fold Change	p-value
Dr.118570	*LOC561493*	*similar to src homology 2 domain containing e*	315.3	1.8E-196
**Dr.133475**	***LOC100151130***	***similar to rasip1 protein***	**254.8**	**3.62E-10**
Dr.85502	*mrc1*	*mannose receptor c1-like protein*	225.7	4.0E-20
Dr.47591	*etv2*	*ets variant gene 2*	219.0	4.9E-237
Dr.84866	*LOC100005159*	*similar to hemicentin 1*	159.4	9.6E-04
**Dr.152900**	***LOC572378***	***similar to transforming growth factor-b type ii receptor***	**110.1**	**4.90E-03**
Dr.107226	*sc:d0254*	*sc:d0254*	37.9	5.0E-10
Dr.78408	*fli1a*	*friend leukemia integration 1a*	37.6	3.0E-61
Dr.86352	*zgc:113016*	*zgc:113016*	36.3	1.5E-09
Dr.36543	*aqp8a*	*aquaporin 8a*	32.6	4.9E-237
Dr.83871	*rasgrp3*	*ras guanyl releasing protein 3 (calcium and dag-regulated)*	32.6	2.6E-08
**Dr.89996**	***egfl7***	***egf-like-domain, multiple 7***	**21.6**	**1.5E-10**
Dr.79866	*yrk*	*yes-related kinase*	20.9	7.0E-151
Dr.103153	*plxnd1*	*plexin d1*	20.1	4.9E-237
Dr.118013	*cdh5*	*cadherin 5*	19.1	3.5E-18
Dr.80968	*fli1b*	*friend leukemia integration 1b*	16.3	1.8E-07
Dr.77989	*dusp5*	*dual specificity phosphatase 5*	14.8	1.2E-16
**Dr.76027**	***ker18***	***keratin 18***	**12.5**	**2.1E-05**
Dr.75812	*tal1*	*t-cell acute lymphocytic leukemia 1*	12.4	6.9E-08
**Dr.150623**	***f10***	***coagulation factor x***	**9.8**	**7.2E-04**
Dr.89035	*tmem88a*	*transmembrane protein 88 a*	9.2	7.2E-16
Dr.75094	*kdrl*	*kinase insert domain receptor like*	7.8	1.6E-36
Dr.80363	*clec14a*	*c-type lectin domain family 14, member a*	6.3	4.0E-06
Dr.151971	*sox7*	*sry-box containing gene 7*	6.1	4.4E-02
**Dr.74559**	***scarf1***	***scavenger receptor class f, member 1***	**5.6**	**1.4E-02**
**Dr.75958**	***robo4***	***roundabout homolog 4***	**5.4**	**3.7E-14**
**Dr.87001**	***cldn5b***	***claudin5b***	**5.1**	**1.6E-15**
Dr.83306	*mcam*	*melanoma cell adhesion molecule*	4.7	4.0E-39
Dr.52827	*zfpm2b*	*zinc finger protein, multitype 2b*	4.4	1.5E-05
Dr.5660	*crip2*	*cysteine-rich protein 2*	4.4	9.4E-11
**Dr.107483**	***sb:cb911***	***sb:cb911***	**4.3**	**3.1E-03**
Dr.75385	*LOC563577*	*similar to novel apoptosis-stimulating protein of p53*	4.2	3.1E-06
**Dr.132454**	***smox***	***spermine oxidase***	**3.2**	**4.8E-55**
Dr.599	*ldb2a*	*lim-domain binding factor 2a*	3.2	3.7E-03
Dr.81683	*rbpms2*	*rna binding protein with multiple splicing 2*	3.1	1.2E-09
Dr.81298	*flt4*	*fms-related tyrosine kinase 4*	3.0	2.1E-29
**Dr.79626**	***ildr2***	***immunoglobulin-like domain containing receptor 2***	**2.9**	**1.4E-21**
**Dr.132331**	***nrp1b***	***neuropilin 1b***	**2.9**	**2.5E-15**
**Dr.78142**	***acvrl1***	***activin a receptor type ii-like 1***	**2.8**	**6.7E-19**
Dr.135121	*stab2*	*stabilin*	2.7	4.5E-10
Dr.76054	*tpm4*	*tropomyosin 4*	2.6	1.9E-37
**Dr.79413**	***jam2***	***junctional adhesion molecule 2***	**2.6**	**1.5E-21**
Dr.91385	*kdr*	*kinase insert domain receptor (a type iii receptor tyrosine kinase)*	2.5	2.5E-29
Dr.88777	*pdlim4*	*pdz and lim domain 4*	2.5	2.3E-02
Dr.76395	*C8orf4*	*chromosome 8 open reading frame 4*	2.4	1.0E-13
Dr.37960	*fgfrl1b*	*fibroblast growth factor receptor-like 1b*	2.4	2.1E-07
Dr.75409	*gapdhs*	*glyceraldehyde-3-phosphate dehydrogenase, spermatogenic*	2.3	2.7E-03
**Dr.22604**	***amot***	***angiomotin***	**2.3**	**2.4E-08**
Dr.80539	*elovl1b*	*elongation of very long chain fatty acids-like 1b*	2.3	2.7E-09
Dr.82429	*LOC563907*	*similar to tumor endothelial marker 8*	2.1	6.5E-03
Dr.104822	*si:dkey-261h17.1*	*si:dkey-261h17.1*	2.1	4.6E-107
Dr.78553	*micall2*	*mical-like 2*	2.0	5.6E-18

Genes in bold text were identified within the *etsrp* overexpressed dataset by visual inspection of the data. Those not in bold were identified by DAVID cluster analysis.

In addition to endothelial specific genes, there were 4 genes of the myeloid lineage that were preferentially induced by *etsrp* oe. They are *lplastin*, *csf3r, cebp1, hsd3b7*. This observation is consistent with what has been previously reported by our group [6]. Overall using the DAVID GO program and visual inspection of the RNA-seq dataset we observed that a substantial number of genes (6.2%) are associated with developing endothelial biology, confirming the biological relevance of the upregulated dataset. Contrarily, DAVID GO analysis of the downregulated genes did not result in a distinct biological category of genes, as genes associated with the development of all three germ layers including endoderm, mesoderm, and ectoderm were repressed.

**Figure 2:**
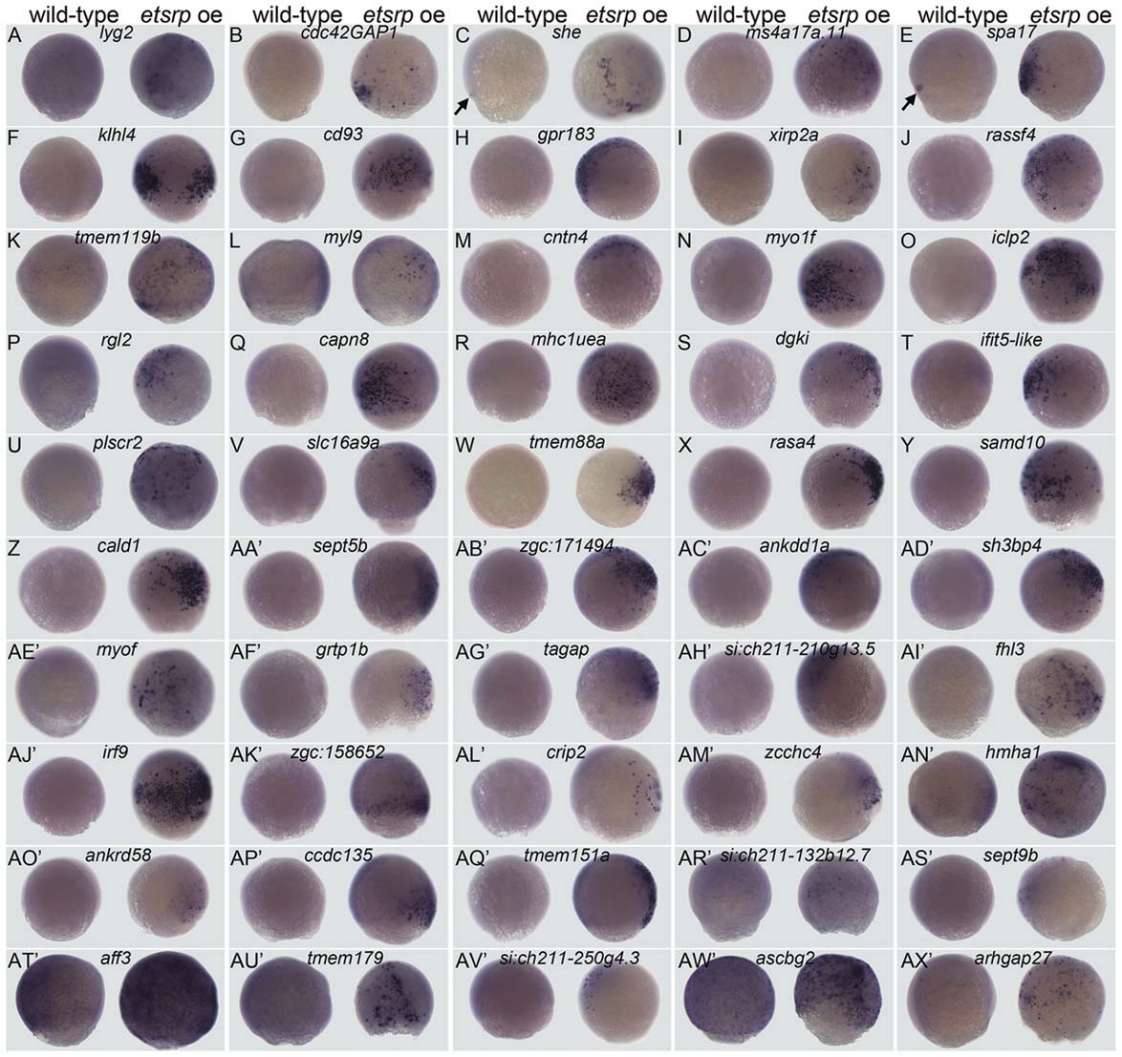
Verification of RNA-seq data. Wild type and *etsrp* injected, *etsrp* oe, embryos were collected at late stages of gastrulation and processed by WISH. Ectopic induction was detected as random positively labeled cells in embryos injected with *etsrp* RNA at the one cell stage. Endogenous expression was observed in the presumptive forerunner cells (arrows) of wild-type uninjected embryos in (C) *she* and (E) *spa17*. Endogenous expression was also observed in (K) *tmem119b* (L) *myl9* (AD′) *sh3bp4* (AN′) *hmha1* (AT′) *aff3* and (AW′) *acsbg2*. Ectopic induction is clearly detected in 49 of the 50 genes examined, but not (AT′) *aff3*, which has a relatively high level of endogenous expression at this stage. Wild type uninjected embryos are positioned on the left column with their *etsrp* oe counterparts on the right for each gene, anterior is facing left where possible. Scale bar: 250 µm.

In order to further biologically validate the data and identify new genes expressed in endothelial cells, several genes were selected from the upregulated dataset for analysis by RNA whole mount in situ hybridization (WISH) at the 80% epiboly stages, when they are predicted to be upregulated by overexpressing *etsrp* (Figure 2). For gene selection, the potential of the top 350 hits to be expressed in endothelial cells was evaluated thoroughly, while the remaining portion was assessed at random. Genes examined were selected based on a PUBMED literature search using the following criteria: 1. Not previously shown to be expressed in vascular endothelial cells. 2. Previously detected in endothelial cells but evidence is limited to *in vitro* studies. 3. Has not been examined in developing vasculature. One of the top ranked genes (highest fold-induction), *similar to Src homology 2 domain containing E*, *she*, was used as a positive control since its expression in the vasculature has been reported previously [19]. The gene set examined is listed on Table 3. This assay resulted in the clear ectopic induction by *etsrp* overexpression in 49 of the 50 genes examined (Figure 2). The high percentage of validated genes induced by *etsrp* overexpression by WISH supports the validity of both the deep sequencing and bioinformatic methods used to examine the transcriptional profiles of upregulated genes. The genes predicted to be suppressed by this approach was also evaluated by quantitative RT-PCR, but only 2of 8 genes examined were validated (Table S3). This suggests that this approach is more amenable to the examination of gene induction by *etsrp*. Nevertheless, the complete dataset containing downregulated unigene entries is listed in Table S8.

**Table 3:** Genes examined from the RNA-seq *etsrp* overexpression dataset.

Unigene ID	Gene ID	Gene Description	Fold Change	p-value
**Dr.153782**	***lyg2***	***lysozyme G like 2***	**1899.9**	**4.5E-26**
**Dr.149118**	***cdc42GAP1***	***similar to Cdc42 GTPase-activating protein***	**441.5**	**1.9E-21**
**Dr.118570**	***she***	***similar to Src homology 2 domain containing E***	**315.3**	**1.8E-196**
Dr.40434	*ms4a17a.11*	*membrane-spanning 4-domains, subfamily A, member 17A.11*	300.4	3.5E-06
**Dr.84343**	***spa17***	***sperm auto antigen 17***	**225.9**	**1.1E-09**
**Dr.103328**	***klhl4***	***kelch like 4***	**198.7**	**2.0E-05**
**Dr.110713**	***LOC100149611***	***similar to LOC495463 protein ;*** ** “** ***CD93”***	**165.4**	**3.8E-08**
**Dr.111220**	***gpr183***	***g protein-coupled receptor 183***	**151.4**	**1.9E-04**
**Dr.78041**	***xirp2***	***xin actin-binding repeat containing 2***	**95.0**	**7.6E-56**
**Dr.134501**	***rassf4***	***ras association (RalGDS/AF-6) domain family member 4***	**51.4**	**1.4E-02**
**Dr.91332**	***tmem119b***	***transmembrane protein 119***	**50.7**	**1.8E-42**
**Dr.89765**	***myl9***	***myosin, light chain 9, regulatory***	**50.1**	**5.7E-113**
**Dr.115399**	***cntn4***	***contactin 4***	**38.4**	**1.4E-02**
**Dr.84022**	***myo1E***	***similar to myosin IE***	**37.9**	**8.4E-03**
**Dr.75719**	***iclp2***	***invariant chain-like protein 2***	**32.2**	**1.3E-16**
**Dr.88587**	***rgl2***	***ral guanine nucleotide dissociation stimulator-like 2***	**28.2**	**1.9E-36**
**Dr.76656**	***capn8***	***calpain 8***	**23.5**	**1.1E-05**
**Dr.11010**	***mhc1uea***	***major histocompatibility complex class I UEA gene***	**21.8**	**2.4E-21**
**Dr.77849**	***dgki***	***similar to diacylglycerol kinase, iota***	**17.4**	**4.4E-04**
**Dr.117215**	***ifit5***	***similar to Interferon-induced protein with tetratricopeptide repeats 5***	**15.1**	**2.1E-03**
Dr.134371	*plscr2*	*similar to phospholipid scramblase 2*	10.6	1.5E-47
Dr.7340	*slc16a9a*	*solute carrier family 16 (monocarboxylic acid transporters), member 9a*	9.3	2.1E-23
**Dr.89035**	***tmem88a***	***transmembrane protein 88a***	**9.2**	**7.2E-16**
**Dr.100033**	***rasa4***	***ras p21 protein activator 4-like***	**7.8**	**2.3E-04**
**Dr.96217**	***samd10***	***similar to sterile alpha motif domain containing 10***	**7.2**	**1.2E-02**
**Dr.114623**	***cald1***	***caldesmon 1***	**6.8**	**1.6E-22**
**Dr.91020**	***sept5b***	***septin 5b***	**6.7**	**1.3E-05**
**Dr.119058**	***zgc:171494***	***zgc:171494***	**6.4**	**2.8E-02**
**Dr.84654**	***ankdd1a***	***ankyrin repeat and death domain containing 1A***	**6.4**	**2.5E-09**
**Dr.91634**	***sh3bp4***	***novel protein similar to vertebrate SH3-domain binding protein 4***	**6.0**	**2.0E-06**
**Dr.82145**	***myof***	***similar to fer-1-like 3, myoferlin***	**5.7**	**4.1E-32**
**Dr.84960**	***grtp1b***	***growth hormone regulated TBC protein 1b***	**5.6**	**1.4E-02**
**Dr.85673**	***tagap***	***t-cell activation GTPase activating protein***	**5.5**	**2.8E-03**
Dr.124255	*plc-l2*	*similar to Inactive phospholipase c-like protein 2*	5.2	2.9E-05
**Dr.80073**	***fhl3***	***four and a half LIM domains 3***	**5.1**	**5.4E-37**
**Dr.133138**	***irf9***	***interferon regulatory factor 9***	**4.9**	**9.4E-12**
Dr.18530	*FAM166B*	*family with sequence similarity 166, member B*	4.4	1.8E-04
Dr.5660	*crip2*	*cysteine-rich protein 2.*	4.4	9.4E-11
Dr.135601	*zcchc4*	*zinc finger, CCHC domain containing 4*	4.0	4.4E-03
**Dr.77065**	***hmha1***	***histocompatibility (minor) HA-1***	**4.0**	**9.1E-12**
**Dr.47691**	***ankrd58***	***ankyrin repeat domain-containing protein 58-like***	**3.8**	**3.1E-06**
**Dr.92393**	***ccdc135***	***similar to coiled-coil domain-containing protein 135***	**3.6**	**5.2E-05**
**Dr.83578**	***tmem151b***	***similar to transmembrane protein tmem151B***	**3.5**	**1.9E-03**
Dr.81141	*si:ch211-132b12.7*	*hypothetical protein LOC564531*	3.5	1.4E-02
Dr.78875	*sept9b*	*septin 9b*	3.3	1.3E-05
**Dr.83297**	***aff3***	***af4/fmr2 family, member 3; LAF***	**2.4**	**1.5E-10**
Dr.90997	*tmem179*	*transmembrane protein 179*	2.0	2.8E-09
**Dr.34190**	***si:ch211-250g4.3***	***si:ch211-250g4.3***	**2.0**	**4.5E-04**
**Dr.107097**	***acsbg2***	***Acyl-CoA synthetase bubblegum family member 2***	**1.9**	**2.8E-64**
**Dr.77920**	***arhgap27***	***similar to Rho GTPase-activating protein 27***	**1.9**	**9.1E-07**

Bold are expressed in vascular endothelial cells at the time examined (24 hpf).

**Figure 3:**
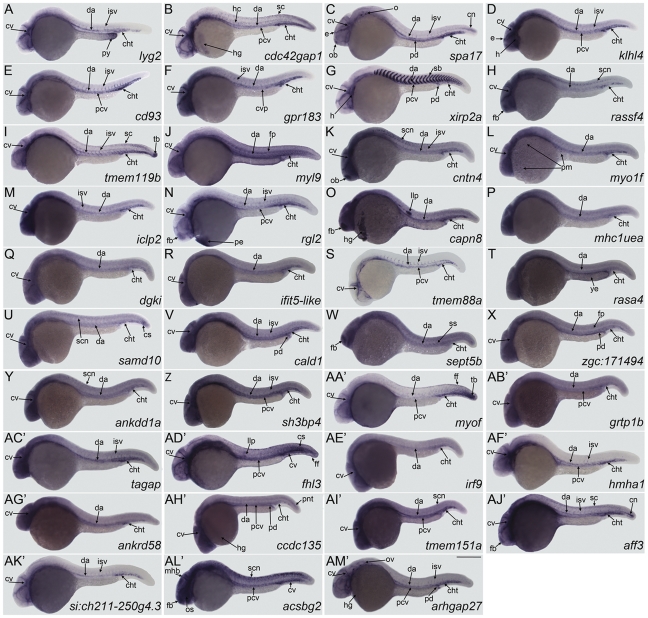
Genes with expression in vascular endothelial cells. The following genes are restricted to the vasculature and are expressed in the cranial vasculature, dorsal aorta, caudal hemtopoietic tail region, posterior cardinal vein, and intersegmental vessels: (E) *cd93*; (F) *gpr183*; (S) *tmem88a*; (AF′) *hmha1*, while the following are expressed in the same structures but exclude the posterior cardinal vein: (A) *lyg2* is also expressed in the posterior yolk extension; (AC′) *tagap*; (AK′) *si:ch211-250g4.3*. These genes are expressed in the cranial vasculature, dorsal aorta, caudal hematopoietic tail region, posterior cardinal vein and other tissues as noted: (B) *cdc42gap1*, is also expressed in the hypochord, hatching gland, and sparsely in spinal cord; (D) *klhl4* is also expressed in the heart, intersegmental vessels, and epiphysis; (G) *xirp2a* is also expressed in the heart, somite boundaries and pronephric duct; (N) *rgl2*, is also expressed in the intersegmental vessels, forebrain and primitive erythorcytes; (AA′) *myof* is also expressed in the fin fold, and tailbud; (AM′) *arhgap27* is also expressed in the intersegmental vessels, otic vesicles, pronephric duct, and hatching gland. The following genes are expressed in the cranial vasculature, dorsal aorta, caudal hematopoietic tail region, and intersegmental vessels as well as (C) *spa17*, the olfactory bulb, epiphysis, otoliths, pronephric duct, and caudal notocord; (H) *rassf4* was not detected in the intersegmental vessels but is observed in the forebrain, floor plate of neural tube, and spinal cord neurons; (I) *tmem119b* is also expressed in spinal cord, and tailbud; (AJ′) *aff3* is also expressed in the forebrain, spinal cord, and caudal notocord. (AD′) *fhl3* is expressed in the cranial vasculature, posterior cardinal vein, caudal vein, fin fold, lateral line primordium, and lightly in caudal somites. (AL′) *acsbg2* is expressed in the posterior cardinal vein, caudal vein, spinal cord neurons, optic stalk, forebrain, and midbrain-hindbrain boundary. The following genes have basal levels of ubiquitous expression with prominent labeling of the cranial vasculature, dorsal aorta, caudal hematopoietic tail region and other structures and tissues where indicated: (J) *myl9* is also expressed in the floor plate; (K) *cntn4*, is also expressed in the intersegmental vessels, olfactory bulb, and spinal cord neurons; (L) *myo1f* is also expressed in primitive myeloid cells; (M) *iclp2*, also has slight expression in anterior intersegmental vessels; (Q) *dgki*; (R) *ifit5-like*; (U) *samd10* is also expressed in the caudal somites, and neurons of the anterior spinal cord; (V) *cald1* is also expressed in the intersegmental vessels and pronephric duct; (X) *zgc:171494* is also expressed in the floor plate, and pronephric duct; (Y) *ankdd1a* is also expressed in the spinal cord neurons; (Z) *sh3bp4* is also expressed in the posterior cardinal vein, and intersegmental vessels; (AB′) *grtp1b* is also expressed in the posterior cardinal vein; (AE′) *irf9*; (AG′) *ankrd58*; (AH′) ccdc135 is also expressed in the posterior cardinal vein, pronephric duct, posterior neural tube, and slightly in the hatching gland; (AI′) *tmem151a* is also expressed in the posterior cardinal vein, spinal cord neurons and intersegmental vessels (see Figure S1AI′). Basal ubiquitous expression with darker staining of the dorsal aorta and caudal hematopoietic tail region is noted in: (O) *capn8* which includes expression in the forebrain, hatching gland, and lateral line primordium; (P) *mhc1uea*; (T) *rasa4* is also expressed in the yolk extension; (W) *sept5b* is also expressed in the forebrain and somites. Embryos are positioned laterally with the anterior facing left. Abbreviations: cht, caudal hematopoietic tail region; cn, caudal notochord; cs, caudal somites; cv, cranial vasculature; da, dorsal aorta; e, epiphysis; ff, fin fold; fp, floor plate; h, heart; hg, hatching gland; isv, intersegmental vessels; llp, lateral line primordium; mhb, midbrain hindbrain boundary; o, otoliths; ob, olfactory bulb; os, optic stalk; ov, otic vesicle; pd, pronephric duct; pe, primitive erythroid cells; pm, primitive myeloid cells; py, posterior yolk extension; sb, somite boundaries; sc, spinal cord; scn, spinal cord neurons; ss, somites; tb, tailbud; ye, yolk extension. Scale bar: 250 µm.

**Figure 4:**
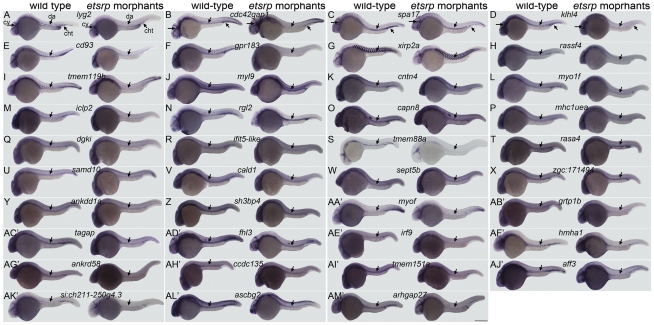
Analysis of gene expression in *etsrp* morphants. *Flk1-gfp* embryos were injected at the one cell stage with a mixture of translation blocking morpholinos for *etsrp* and analyzed by WISH at the 24hpf stage. Marked reduction is apparent in all genes examined in the axial vasculature, which includes the dorsal aorta and posterior cardinal vein, and is marked with a downward facing arrow in all images. The primitive myeloid cells stained in (L) *myo1f* wild-type controls are absent in their *etsrp* morphant counterparts. The staining in the axial trunk region of *rgl2* in *etsrp* morphants marks primitive erythrocytes that are trapped due to lack of circulation. The expression of non-vascular structures is not affected otherwise in *etsrp* morphants. Embryos are positioned laterally with the anterior facing left. Abbreviations: cht, caudal hematopoietic tail region; cv, cranial vasculature; da, dorsal aorta. Scale bar: 250 µm.

To determine whether the 50 selected ectopically induced genes are expressed in the developing embryonic zebrafish vasculature, embryos were processed for WISH at a developmental stage when most of the primitive vasculature has formed, 24-hour post fertilization (24hpf). Marked expression in the vasculature was noted for 39 of the 50 genes (Figure 3). To confirm that these genes are functionally downstream of *etsrp* in the vasculature, we examined their expression in *etsrp* morphants, in which case the expression in the axial vasculature is prominently reduced as demonstrated in Figure 4. Sparse expression in myeloid cells was only detected for one of these genes, *myo1F* (Figure 3L). Although there is some ubiquitous expression of some of these genes, the more pronounced expression in the vasculature is clearly demonstrated in higher magnification images of the trunk regions (Figure S1), where the axial vascular expression in morphants is clearly reduced in all genes. Of the 39 genes with vascular expression only *fhl3* (Figure 3 AD′ and Figure S1AD′) and *acsbg2* (Figure 3AL′ and Figure S1AL′) are preferentially expressed in the axial venous system at this stage.

In the course of gene selection and WISH probe generation the Ensembl databases were utilized together with those at NCBI to examine gene structure and evolutionary ontology. Several of the genes probed for were noted to have redundant gene names as of the current genome build, Zv9 in Ensembl. These paralogs have been noted in their description below with an asterisk and further depicted in Table S4 with chromosomal location and identity. Following is a brief description of 10 selected genes in the order that they are presented in Figures 3 and 4. Descriptions for the remaining 29 genes are continued in Text S1.

### A. *lyg2*

The goose type lysozyme ortholog *lyg2* was originally identified in goose egg white, where it functions in the catalysis of bacterial cell wall break down, and is expressed ubiquitously in flounder [29]. In chicken, it is expressed in lung and non-adherent fraction of bone marrow cells, possibly reflecting expression within myelocytes where it exhibits an innate immune function [30]. Contrary to mammalian *lysozyme G*, the *lysozyme G2* in flounder and zebrafish lack a signal peptide and a conserved cystein catalytic triad, suggesting an intracellular role with a potentially distinct function [31]. There are 2 other paralogs for this gene. *lyg1* (*zgc:92608*), is expressed in macrophages [32], while the expression of *zgc:162941* has not been reported.

### B. *cdc42GAP1/ARHGAP1*

Originally cloned in mice, *cdc42GAP1* catalyzes the hydrolysis of GTP bound by the Rho GTPases cdc42 and rac1, inactivating them [33]. In mice and humans it is expressed ubiquitously but exhibits marked expression in heart and lungs [34]. Mice knockouts result in generally reduced organ size relative to wild-type siblings due to increased rates of basal apoptosis, and display reduced viability [35]. The few survivors experience premature aging associated with heightened genomic instability [36]. In vitro evidence suggests *cdc42GAP1* negatively modulates angiogenesis [37]. Although it is expressed ubiquitously in mammals, it is predominantly expressed in the developing vasculature and heart in zebrafish at the stage examined here (Figure 3B).

### C. *spa17/zgc:153721*

The protein is homologous with the human *sperm protein antigen 17*, *spa17*, and both contain a cAMP dependent protein kinase regulatory subunit domain and an IQ motif that binds proteins with EF-hand motifs, but *zgc:153721/spa17* of zebrafish has a glutamine rich region between these motifs that is absent in *spa17* in humans and mice. Although in mice it is expressed ubiquitously and is expressed in embryos, in rabbits, it is most highly expressed in the tissue from which it was originally identified, testis [38], [39]. Increased expression of *spa17* has been detected in esophageal, ovarian, and cervical cancers and has been used as a clinical marker for cancer in these tissues [40], [41], [42]. In zebrafish, *spa17* is also expressed in the pronephros (Figure 3C).

### D. *klhl4*

*kelch like homolog like 4*, *klhl4* is a member of the kelch motif containing family of proteins [43]. The founding member, *kelch*, was cloned from Drosophila, and mediates cytoplasmic streaming through ring canals in the developing oocyte [44]. In human fetal tissue, *klhl4* shows ubiquitous expression, and the protein is structurally similar to the founding member, *kelch1* [45]. As in humans, the *klhl4* of zebrafish has a BTB/POZ like domain and a BACK domain at the N-terminus, but while human *klhl4* has 6 kelch domains at the carboxy terminus, zebrafish has 3. The function of this protein has not been characterized.

### E. *cd93*

Other aliases of *cd93* include *complement component C1q receptor like* (*C1qrl*), *lymphocyte antigen 68* (*ly68*), and *AA4.1*. *cd93* is a widely conserved member of the c type lectin family whose expression in endothelial and other cell types has been documented in mice and humans [46], [47]. In previous reports the name *complement receptor C1qR-like* (*crl*) has been used for a different gene in zebrafish, which is the likely homolog of *c-type lectin domain family 14*, *member a* (*clec14a*) [3], [18], [19]. Although both of these genes are members of the c-type lectin family, they are paralogs of each other that deserve distinction, and we note that both are expressed in the vasculature, since *clec14a* [3] and *cd93* shown here, are vascular specific at the stage examined (Figure 3E).

### F. gpr183*

*Epstein bar induced 2, ebi2*, or *g protein coupled receptor 183, gpr183*, is an orphan receptor that was originally identified as a gene induced by Epstein Bar Virus infection in Buritt lymphoma cells [48]. Knockout mice are viable without any notable gross phenotype, however they experience defects in B-cell mobility within lymphoid tissues, and consequently have difficulty mounting rapid antibody responses [49], [50]. Northern blot evaluation of its expression was originally limited to peripheral blood mononuclear cells (PBMCs), lymphocytic tissues and lungs [48]. However, in separate assays it has been detected in the aorta, atria, gastrointestinal tract, but not in the hematopoietic bone marrow or fetal liver [51]. At the stage examined, *gpr183* is clearly expressed in the developing vasculature of zebrafish embryos (Figure 3F), but not in the heart.

### G. *xirp2a*

*xin actin binding repeat containing 2a*, *xirp2a*, is one of three orthologs for the mammalian counterpart, *xirp1β* [52]. The paralogs in zebrafish are *xirp2b*, which is restricted to the myotome borders during the larval stages of development (ZFIN), and *xirp2c*, which has not been characterized. Functionally, xin repeat motifs cross-link f-actin with β-catenin at adherens junctions [53]. *Xirp2a* expression in the myoseptum, pronephric duct, heart, otic vesicle, head mesoderm, and liver has been demonstrated in developing zebrafish larvae [32]. In this study the expression in cranial and axial vasculature in the trunk becomes more evident. The most likely explanation for the differences observed might be a longer in situ color developing time used here.

### H. *rassf4*

*ras association (RalGDS/AF-6) domain family member 4*, *rassf4*, is a member of Ras association domain family of proteins, which contain a Ras association domain and in the case of *rassf4*, a SARAH domain (reviewed in [54]). *rassf4* is expressed ubiquitously in humans, but is downregulated in tumorous cells due to the hypermethylation of its promoter [55]. The idea that *rassf4* is a tumor suppressor is supported by the apoptotic death of tumor cells following forced expression of *rassf4* [55]. Besides expression in the vasculature of 1 dpf zebrafish embryos, *rassf4* is also highly expressed in spinal cord neurons (Figure 3H, and Figure S1H).

### I. *tmem119b*

Neither the protein encoded by *tmem119b* nor its paralog, *tmem119a*, have been characterized. *tmem119b* encodes a single pass type I transmembrane protein while *tmem119a* encodes a multipass transmembrane protein with two transmembrane domains. As a conserved gene in vertebrates, it is currently unclear whether *tmem119b* or *tmem119a* is the ortholog of their counterpart in mice, *osteoblast induction factor*, *obif*, which promotes osteoblast differentiation [56]. Although *obif* and *tmem119b* share a structural topological profile, *tmem119a* has a negligible higher amount of amino acid conservation with *obif*. It is also currently unknown whether *tmem119*/*obif* plays the same roles in humans.

### J. *myl9*

*myosin light chain 9 regulatory*, *myl9*, is a calcium-regulated protein that regulates the contraction of myosin heavy chains in non-skeletal muscle cells that was originally cloned from human umbilical artery [57]. Currently there is evidence that the expression of *myl9* may be directly regulated by *junb* [58], and *runx1* [59]. In zebrafish there is a close paralog, *myosin light polypeptide 9-like* (*zgc: 77916*), with 95% gene identity to *myl9* that also remains to be characterized.

## Discussion

In this report we provide further support for the utility of combining the relatively unbiased approach of high throughput next generation RNA-sequencing to analyze whole embryo transcriptomes in zebrafish. The high correlation between genes induced by *etsrp* as predicted by RNA-seq and their confirmation by WISH reinforces the effectiveness of the methods used to interrogate transcriptional profiles in zebrafish. Of the genes selected from the dataset obtained, we identified a very high percentage of genes expressed in the vasculature of embryos, validating this approach to identify biologically relevant genes. However there may be non-vascular genes in this data that are induced due to the induction of off-target genes by the artificially elevated level of *etsrp*. This issue can be addressed by WISH using wild type embryos at a proper embryonic stage. Additionally, this method does not discriminate direct and indirect targets induced by *etsrp*. Further analysis by ChIP-Seq should reveal those genes that are transcriptionally regulated by *etsrp*.

Both the ectopic induction of these genes by *etsrp* overexpression and down-regulation by *etsrp* deficiency in vascular endothelial and primitive myeloid cells highlights their genetic epistatic relationship to *etsrp* and show that *etsrp* is a potent transcriptional activator whose expression and activity must be tightly regulated. Although several genes exhibit basal levels of ubiquitous expression their level in the vasculature is still higher. These genes deserve as much attention as those only specific to the vascular lineage, as several studies have shown that the down-regulation of such genes resulted in the disruption of vascular development. For instance, knockdown of *nrarp-a* and *nrarp-b* [60], [61], *lpa_1_* [62], and *dep1a*/*dep1b* [63] respectively, resulted in defects in intersegmental vessels, lymphangiogenesis, and arterial specification. We obtained approximately 850 genes that were induced 1.9 fold or more by *etsrp* expression. Based on previous annotation and studies we can classify 6.2% of them as vascular genes. Experimental studies identified 39 more vascular genes and further increased the percentage to 10.8%. This leaves over 700 unigene entries in the dataset, some of which may be expressed and actively involved in the development of the zebrafish vasculature, and are potentially worth examining. The complete dataset is available in Table S5 and we hope this will benefit research on the development of the vascular system. The panel of genes found here remain to be characterized further and will require subsequent functional studies.

## Materials and Methods

### Embryo injection

*Flk1-gfp* embryos were injected with 75 pg *etsrp* RNA at the one cell stage as described [5], and collected at the late gastrulation stages, 70–90% epiboly, when ectopic *flk1-gfp* expression is notable in injected embryos. Total RNA was extracted with the PerfectPure RNA Tissue kit (5 Prime). For pre-sequencing validation, cDNA was made with Oligo(dT) primers and Superscript II reverse transcriptase (Invitrogen). The following primers pairs were used for verification by RT-PCR: *β-actin*: 5′ -TGTTTTCCCCTCCATTGTTG-3′, 5′-ACATACATGGCAGGGGTGTT-3′; *etsrp 3′UTR*: 5′-GAGGAATTCTCGAAGGATTGG-3′;TGGTTTTCTAAAGGCACCTAG-3′; *Fli1a*: 5′-CCGAGGTCCTGCTCTCACAT-3′;5′-GGGACTGGTCAGCGTGAGAT-3′; *scl*: 5′-GGAGATGCGGAACAGTATGG-3′, 5′-GAAGGCACCGTTCACATTCT-3′. To test gene expression in *etsrp* morphants, *flk1-gfp* embryos were injected with a mixture of 4 ng each of translation blocking *etsrp* morpholinos as described in [5] at the one cell stage, and embryos were harvested at 24hpf for WISH.

### Cloning and Whole Mount In Situ Hybridization (WISH)

Different segments of the genes selected for evaluation were cloned into the pCRII Topo vector with the TOPO TA cloning kit (dual promoter) according to the manufacturer's instructions (Invitrogen). Amplicons were obtained from an *etsrp* overexpression cDNA library at late stages of gastrulation (70%–90% epiboly). Primers used for amplification are noted in Table S5. Positive plasmids were confirmed by sequencing. WISH was performed as described [64]. DIG labeled RNA probes were generated by linearizing TOPO-cloned genes with restriction endonucleases (New England Biolabs), and transcribing with SP6 or T7 RNA polymerase (Promega).

### Image acquisition and processing

In situ stained embryos were further processed by a serial dehydration in ethanol, followed by rehydration into 1XPBS. Embryos were imaged in 2% methylcellulose in depression microscope slides. Images were captured with a color digital CCD camera (Axiocam, Zeiss) mounted on an upright microscope (Axioskop2 plus, Zeiss) with Openlab 4.0.2 software (Improvision, Lexington, MA). Serial images were combined, merged and processed with Adobe Photoshop CS5.

### Deep sequencing library construction and bioinformatics

mRNA-seq libraries were constructed with the Tru-Seq RNA prep kit (Illumina) according to manufacturer's instructions. After this project was initiated, the Illumina sequencer has gone through a series of improvements. As a result, we obtained both single-end and paired-end reads with different lengths (76, 51, and 46 bp) using the Illumina GA II sequencer and its earlier versions. The obtained reads were mapped to 51,481 Unigene transcript sequences (Build #117, October 2009) [26] using BWA [27] allowing up to 4 mismatches. In case of the paired-end reads, we determined whether the mapped results support correct pairing of the reads according to the unigene annotation. If one read in a pair is mapped to a gene, the other one should be mapped to the same gene. Since each read in a pair may map to multiple locations in the genome, all possible combinations of their mappings were examined for correct pairing. The pair of reads is considered uniquely mapped only if one unique pair of mapped locations was identified.

To determine expression levels of genes and exons, we used the variable RPKM (reads per kilobase of exon per million mapped reads) defined by Mortazavi et al. 2008 [65]. Analysis of differential gene expression was carried out to obtain the differentially expressed genes between control sample and *estrp* oe sample. The number of uniquely mapped read-pairs for each gene in each sample was stored. The total number of mapped reads in each lane was normalized using the total mapped reads in each lane. Fisher's exact test was then performed using the above read counts for each gene. The resulting p-values were corrected via the Benjamini and Hochberg method as implemented in R. Finally, differentially expressed genes were defined as those with changes of at least 1.9-fold between a pair of samples at a false discovery rate (FDR) of 5%. Table S7 lists all 849 upregulated unigene hits in the *estrp* oe sample, while the 726 downregulated hits are tabulated in Table S8.

### Quantitative PCR (qPCR)

qPCR was performed and analyzed exactly as stated in [12], with three independent biological replicates of either uninjected or *etsrp* oe groups of embryos harvested at late gastrulation stages. Primers used are listed in Table S6.

## Supporting Information

Figure S1 **Higher magnification of** **Figure 4** **.** The axial trunk vasculature of embryos displayed in figure 4 were imaged at higher magnification to highlight the changes observed. Wild-type embryos are on the left half of each column with their *etsrp* morphant counterparts on the right for each gene. Embryos were positioned with anterior facing left.
(TIF)

Table S1 **Total RNA-Seq reads obtained in control and** ***estrp*** **oe samples and mapping results.**
(XLS)

Table S2 **DAVID GO derived gene subset without clear vascular expression or not expressed in developing vasculature.**
(XLS)

Table S3 **Quantitative PCR of unigene hits predicted to be suppressed by** ***etsrp*** **oe via RNA-seq.**
(XLS)

Table S4 **Genes with double entries in Ensembl.**
(XLS)

Table S5 **Primers used for cloning to make WISH probes.**
(XLS)

Table S6 **Primers used to test downregulated gene set following** ***etsrp*** **overexpression by qPCR.**
(XLS)

Table S7 **Complete** ***etsrp*** **mRNA-seq dataset (induced greater than or equal to 1.9 fold by** ***etsrp*** **overexpression).**
(XLS)

Table S8 **Downregulated mRNA-seq dataset (reduced greater than or equal to 1.9 fold by overexpression of** ***etsrp*** **).**
(XLS)

Text S1 **Supplementary Text.** A continuation of the results section that describes information regarding the genes examined in this study.
(DOC)
