# Supplementary figures and images for: Identification of Vascular and Hematopoietic Genes Downstream of etsrp by Deep Sequencing in Zebrafish
Source: PLoS One. 2012 Mar 16;7(3):e31658. doi: 10.1371/journal.pone.0031658 (PMC3306315; doi:10.1371/journal.pone.0031658)

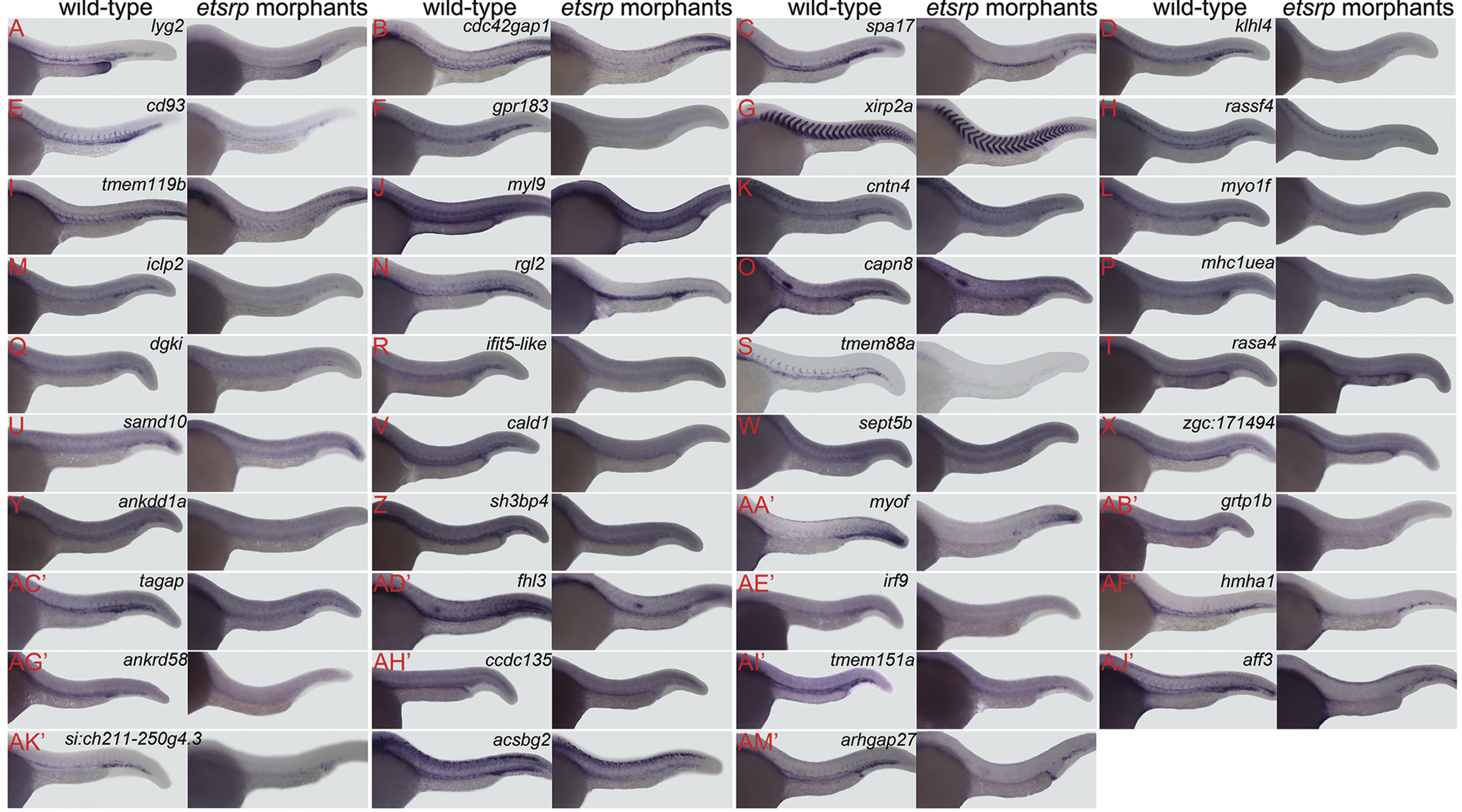

Supplement: Figure S1 — Higher magnification of Figure 4 . The axial trunk vasculature of embryos displayed in figure 4 were imaged at higher magnification to highlight the changes observed. Wild-type embryos are on the left half of each column with their etsrp morphant counterparts on the right for each gene. Embryos were positioned with anterior facing left. (TIF) [file pone.0031658.s001.tif]
