# Supplementary material for: Identification of Vascular and Hematopoietic Genes Downstream of etsrp by Deep Sequencing in Zebrafish
Source: PLoS One. 2012 Mar 16;7(3):e31658. doi: 10.1371/journal.pone.0031658 (PMC3306315; doi:10.1371/journal.pone.0031658)
Supplement: Text S1 — Supplementary Text. A continuation of the results section that describes information regarding the genes examined in this study. (DOC) [file pone.0031658.s010.doc]

**Supplementary Text**

**Text S1**

Genes marked with an asterisk have two identifiers in the current Ensembl build, Zv9. To clarify the identity of the gene examined here, their chromosomal locations have been noted along with their un-examined paralog(s) in Table S3.

K. *cntn4/big2**

*contactin4* is a member of the subset of immunoglobulin superfamily of proteins expressed in the nervous system [1], whose function is crucial for axon guidance in the formation of the olfactory bulbs of mice [2]. In vitro evidence also suggests *contactin4* is involved in axonal migration behaviors during retinotectal development [3]. Robust expression has also been detected in human testis and at lower levels in various other organs [4], but *cntn4* disruption has only been associated with 3p deletion syndrome and autism [5,6]. In zebrafish there is strong *cntn4* expression in the olfactory bulb, spinal chord neurons, and in both axial and cranial vasculature (Figure 3K).

L. *myo1f*

*myo1f* is an unconventional myosin that was initially identified from cochlear tissues of mice and has also been detected in eye, brain, liver, lungs and other tissues [7]. In a separate study, the expression of *myo1F* was only detected in tissues of the immune system, in particular the knockout mice display defects in neutrophil migration due to an inability of neutrophils to exocytose granules that prevent neutrophil adhesion to integrin containing substrates [8]. Of clinical relevance, an association between mutations in *myo1f* and hearing pathologies has been noted [9]. The expression of several unconventional myosins in zebrafish has previously been published [10], and we note that *myo1f* has a low level of ubiquitous basal expression with a more robust expression in vasculature and in myeloid cells (Figure 3L).

M. *iclp2*

The mammalian invariant chain proteins function in antigen presentation by immune cells, however there are two separate genes in zebrafish, *iclp1* and *iclp2*. The latter lacks a fragment that is required for antigen presentation, suggesting that in zebrafish, *iclp2* may have a distinct function from the mammalian invariant chain and this alternate role is believed to be enzymatic in nature [11].

N. *rgl2*

*ral guanine nucleotide dissociation stimulator-like 2*, *rgl2*, is a member of the ral-Guanine Dissociation Stimulator, ral-GDS, family of proteins, which are effectors of ras [12], whose induction by overexpression of *etsrp* was detected previously [13]. *Rgl2* expression in humans is reported to be ubiquitous, as is the mouse ortholog, *rlf* [14,15]. However, *rlf* presents higher expression levels in heart and lung [16]. The activation of *rgl2* by ras proteins is dependent on specific modifications such as phosphorylation by PKA [17], and one function of rgl2 bound by R-ras is the activation of ralA, which promotes exocytosis of endosomes [18]. Of clinical relevance, *rgl2*, promotes hypertrophic growth of terminally differentiated cardiomyocytes [16], and its expression is increased in pancreatic cancer cells, where it promotes cancer cell growth through ral-dependent and ral-independent activation [19]. In zebrafish, *rgl2* is expressed throughout the developing vascular endothelial system, primitive erythrocytes and the forebrain (Figure 3N and Figure S1N).

O. *capn8*

*calpain8* is a member of the calpain family of intracellular calcium regulated cystein proteases, some of whose expression and evolution in developing zebrafish embryos have been previously examined [20]. In mammals *calpain8* is orthologous with *nCL-2*, which is specifically expressed and functional in the stomach of mice [21]. The ortholog in xenopus, *xcl2*, is expressed in non-mesodermal and non-vascular tissues during developmental stages and its knockdown results in gastrulation defects [22]. Nonetheless, we previously detected the upregulation of *calpain8* by overexpressing *etsrp* using a microarray approach [23], and therefore examined its expression here. *Calpain8* of zebrafish has basal ubiquitous levels of expression, with pronounced expression in the axial vasculature, forebrain, lateral line primordium, and hatching gland (Figure 3O).

P. *mhc1uea*

*major histocompatibility class 1 uea* of zebrafish is orthologous with MHC1 Related protein MR1 of humans, which is well conserved and ubiquitously expressed [24]. A current hypothesis is that MR1 presents antigens to immunogenic cells of mucosa associated invariant T cells (mait), the innate T cells resident within the intestine that can mount a rapid immune response, but the antigens presented to mait cells by MR1 are unknown [25]. As previously reported, *mhc1uea* is induced by *etsrp* and expressed ubiquitously [13], and we note here that the expression within the trunk vasculature is reduced in *etsrp* morphants (Figure 4P and Figure S1P).

Q. *dgki*

*diacylglycerol kinase iota*, *dgki*, is one of nine members of diacylglyerol kinases in mammals that are divided into five subtypes, that metabolize and thereby dampen diacylglycerol signaling by phosphorylating it to phosphatidic acid (Reviewed in [26]). The human *dgki* was originally cloned from retinal and brain libraries, and detected in both cytoplasmic and nuclear fractions [27]. *Dgki* knockout mice are viable and appear normal but harbor reduced ras signaling and are disposed to defects in metabotropic glutamate receptor-dependent long-term depression [28,29].

R. *ifit5-like**

*interferon-induced protein with tetratricopeptide repeats 5-like*, may be the ortholog of the mammalian *ifit5*, which was identified in a promyelocytic leukemia cell line, NB4, through differential hybridization as a gene that is induced directly by interpheron alpha and indirectly by retinoic acid [30]. Although knowledge on this gene is limited to the report on its identification, *ifit5* was classified as a member of the interferon inducible family of proteins owing to the means of its induction and the presence of tetratricopeptide repeat (TPR) motifs characteristic of this family. TPR motifs mediate protein-protein interactions, but their functional roles in *ifit5* remains obscure. While human *ifit5* contains 9 TPR motifs [30], a pfam domain scan of the zebrafish *ifit5-like* reported here is predicted to contain 4 TPR motifs.

S. *tmem88a*

*transmembrane protein 88a* is a multipass transmebrane protein with two transmembrane regions. It was previously listed as a gene induced by *etsrp* [13] and its paralog, *tmem88b* has the same topology. While humans have a single ortholog, *tmem88*, mice have two, *tmem88* and *tmem88b*, both of which remain uncharacterized. Like other vascular specific genes, *tmem88a* is downregulated in the axial vasculature of *etsrp* morphants (Figure 3S).

T. *rasa4*

*ras p21 protein activator 4*, *rasa4*, or *Ca2+-promoted ras inactivator*, *Capri*, is a well conserved ras gtpase member of the gap1 family that negatively regulates ras signaling by switching off the Ras-MAPK signaling pathway in response to calcium signaling [31]. By northern blot *rasa4* was found to have ubiquitous expression in humans [32], but knockout mice are viable yet experience an impaired ability to mount innate immune responses to pathogens because it functions as an adaptor for cdc42 and rac during FcR-mediated phagocytosis [33]. Through an RNAi screen, *rasa4* was also identified as one of several hundred genes associated with necropotosis, emphasizing its involvement in tumor signaling inhibition [34]. Although we note a broad distribution of expression at the stage examined, there is bolder expression within the axial vasculature in the trunk of zebrafish embryos that is reduced in *etsrp* morphants (Figure 3T and Figure S1T).

U. *samd10**

The only notable domain in this gene is the sam domain, which is about 70 amino acids long and is heavily credited with mediating protein-protein interactions in a diverse array of proteins and tissues including several proteins that are involved in developmental processes [35]. There is also evidence that sam domains bind mRNA, which increases the possible functional role of the encoded protein (Schultz 2003). We note the expression of *samd10* in both the cranial and axial vasculature and budding somites, but not in intersegmental vessels (Figure 3U).

V. *cald1**

Caldesmon is a protein that binds actin, myosin, tropomyosin and calmodulin. In humans there are two isoforms generated by alternative splicing, the high molecular weight form, *hcald*, is smooth muscle specific and the low molecular weight form, *lcald*, is expressed in non-muscle cells [36]. The main function associated with *caldesmon* is the regulation of cell motility, and is a putative repressor of cancer cell invasion [37,38]. *Lcald* has been identified in cancerous gliomas and in endothelial cells of breast, lung, kidney, colon, stomach, ovary, uterus, prostate, thyroid and liver cancers, but never in the normal vasculature of adults [39]. The *caldesmon1*, *cald1*, of zebrafish is the ortholog of *lcald*, and its knockdown by morpholinos results in heart formation defects but the effects on vascular development remains to be examined [40]. The *cald1* gene examined here is expressed in the vasculature but not in the heart and is located on chromosome 4, while Zheng et al knocked down the cald1 gene located on chromosome 25 (Table S3).

W. *sept5b*

Initially identified as *cell division cycle related 1*, *CDCrel1*, and *peanut like 1*, *PNUTL1*, in a megakaryocytic cell line and endothelial cells [41,42,43], *septin5* expression was also identified in human fetal brain and heart but only in cell bodies and dendrites of adult neurons, and platelets [44,45]. *CDCrel1/sept5* knockout mice appear normal, which may be due to compensation by other septins, of which there are many [46], but they exhibit social and cognitive disorders [47]. Although there is precedent for the expression of *septin5* within huvecs [48], in zebrafish we also note faint but definite expression that outlines the dorsal aorta in the trunk vasculature (Figure 3W and Figure S1W), as well as the forebrain and somites. The nearest evolutionary paralog, *septin5a* has not been examined.

X. *zgc:171494**

The orthologous gene in higher vertebrates is *ceramide synthase 3*, *cers3*, which adds acyl chains to catalyze the synthesis of ceramides containing C18 and longer acyl chains, is expressed in testis and at low levels in the skin of mice [49]. Although there is some slight ubiquitous expression, there is marked expression of *zgc:171494* in the axial vasculature (Figure 3X) and the expression in the cranial vasculature becomes more apparent in *etsrp* morphants (Figure 4X).

Y. *ankdd1a*

*ankyrin repeat and death domain containing 1a*, *ankdd1a* encodes a protein that is 489 amino acids long and is predicted to contain five ankyrin repeats and a c-terminal death domain. Ankyrin repeats mediate protein-protein interactions, and the death domain associated with apoptosis is also found in proteins involved in non-apoptotic molecular functions [50,51]. Although highly conserved, *ankdd1a* has not been characterized and while mice and humans both have a putative paralog, *ankdd1b*, this paralog in zebrafish has currently not been identified or annotated.

Z. *sh3bp4*

*sh3 binding protein 4*, *sh3bp4*, or *transferring receptor trafficking protein*, *TTP*, was cloned from human cornea fibroblasts and is also expressed in pancreas, heart, placenta, kidney, skeletal muscle, liver [52] and retina [53]. Although the protein encoded by *sh3bp4* has been localized at the plasma membrane by two independent groups, there is debate on its localization at the nucleus [53,54]. Functionally, *sh3bp4* is classified as an accessory endocytic protein that specifically internalizes the transferring receptor [54]. Several interaction partners of *Sh3bp4* have been discovered by yeast two hybrid screens including *Ciz1*, *Plekha1*, and *Ttc1*, which regulate signaling and stress responses [55].

AA. *myof* *

*myoferlin* is induced by *etsrp* overexpression [13], and while it is commonly associated with muscle development, its involvement in endothelial cell biology has also been revealed [56,57]. We demonstrate here that its expression in endothelial cells is conserved in zebrafish with signal detected in the cranial and axial vasculature of the trunk, as well as the somitic mesoderm in the tail of 24 hpf embryos (Figure 3AA’).

AB. *grtp1b*

*growth hormone regulated TBC protein 1b*, was originally identified in the transcriptional profiling of cardiomyocytes in response to growth hormone signaling [58]. Widely conserved, its expression in mice is highest in testis, with moderate expression in kidney and liver and lowest in lung, intestine and stomach. Possession of a TBC domain suggests it functions as an activator of the GTPase, Rab.

AC. *Tagap**

*T-cell activation GTPase activating protein*, *tagap*, is a Rho gap that was identified as a gene that is regulated during T cell activation [59]. In mice *tagap* appears to be expressed ubiquitously with an apparent prominence in sperm and although knockouts are viable, they have transmission ratio distortion defects [60]. Nevertheless, of clinical significance, *tagap* is downregulated in T cells of patients with down syndrome [61], and is associated with celiac disease and type 1 diabetes [62,63,64].

AD. *fhl3**

*four and a half lim domains 3*, *fhl3*, contains four LIM domains that mediate protein-protein interactions, and has been localized in both the cytoplasm and the nucleus [65,66]. In the cytoplasm it regulates α-actinin mediated actin bundling where it enhances cell spreading and stress fiber disassembly [65]. In the nuclei of certain blood lineages, *fhl3* is a transcriptional co-repressor of the beta chain of the IgE receptor [66], while in myogenesis it attenuates *myoD* expression [67]. *Fhl3* is also classified as a co-activator in satellite cells where it cooperates with *sox15* in the activation of *foxk1* [68]. Regarding disease, *fhl3* together with paralogs *fhl1* and *fhl2* are downregulated in liver cancer cells where they function as tumor suppressors through tgf-β dependent and independent signaling by modulation of *smad* activity [69].

AE. *irf9*

*interferon regulatory factor 9*, *irf9*, is part of the heterotrimeric transcription factor, *interferon stimulated gene factor 3*, *isgf3*, which includes the binding partners *stat1* and *stat2* [70]. In normoxic endothelial cells, a direct target of *isgf3* and therefore interferon signaling is *hypoxia inducible factor 1α*, *hif1α*, which results in the inhibition of proliferation [70]. *irf9* is highly expressed in breast and uterine cancer cells, and forced expression of *irf9* regulates interferon response genes independently of interferon signaling, resulting in resistance to anti-microtubule agents and cell survival [71]. However, there is little evidence demonstrating that *irf9* can activate transcription when not in the *isg3* complex [72]. Nonetheless, *etsrp* overexpression ectopically induced the expression of *irf9*, which shows marked expression in the axial (Figure 3AE’) and cranial vasculature of *etsrp* morphants (Figure 4AE’). Neither *stat1* nor *stat2* appeared on the dataset of genes induced by *etsrp*.

AF. *hmha1**

*histocompatibility (minor) HA-1*, located on chromosome 22 encodes a protein with a rhoGAP domain, a protein kinase C-like phorbol ester/diacylglerol binding domain, and an FCH domain. The mammalian ortholog is expressed in hematopoietic cells and some tumorous as well as noncancerous lung and hepatic tissues [73,74]. Although minor histocompatibility antigens are clinically relevant in stem cell and organ transplantation in the mediation of transplant rejection/acceptance [75], the expression and presentation by vascular endothelial cells is currently not believed to activate immunologic responses [76]. The function of *hmha1* in the developing endothelial cells of zebrafish remains to be determined.

AG. *ankrd58*

This gene has a modest level of basal expression but has robust axial vascular expression in the trunk (Figure 3AG’). Based on Ensembl predictions, it is conserved and the mammalian ortholog is *ankrd58*. While zebrafish is predicted to contain two ankyrin repeats, the mouse ortholog also has two, while in humans there are three. Ankyrin repeats are involved in protein-protein interactions [77]. This gene awaits further characterization.

AH. *ccdc135*

*coiled-coil domain containing 135*, *ccdc135*, or *spermatogenesis related gene like*, *srgl*, was first cloned from spermatocytes of mice and also detected in spleen, kidney, ovary and thymus [78]. *Ccdc135* was subsequently identified as a gene that is preferentially expressed in ciliated cells such as olfactory sensory neurons [79], and more recently it was reported that the ortholog in *Drosophila melanogaster* is specifically localized to axonemes where it regulates sperm motility [29]. In 24 hpf zebrafish embryos there is slight basal expression throughout the embryos with pronounced expression in the cranial and axial trunk vasculature, pronephros and tailbud (Figure 3AH’).

AI. *tmem151a**

*tmem151a* encodes a protein with four transmembrane domains that is well conserved in vertebrates. There is a second gene in Ensembl with the same name as well as 4 paralogs called *tmem151b*. *tmem151a* remains to be characterized further.

AJ. *aff3*

*AF4/FMR2 family member 3*, *aff3*, was originally identified as a lymphoid nuclear protein with transactivation potential that is related to the AF-4 family of genes [80]. In mice *aff3* is only expressed in thymus, spleen, brain and lungs [80], while in humans it is expressed in the heart, brain, and placenta of adults, as well as fetal tissues [81]. The non-vertebrate ortholog of drosophila, *lilliputian*, controls cell growth and differentiation [82], suggesting *aff3* may regulate cell growth. Consequently, some breast cancer tumor cells have increased expression of *aff3*, which is otherwise not expressed in these organs [83]. Genetically, *aff3* has been associated with acute lymphoblastic leukemia [81], and a predisposition to rheumatoid arthritis [84].

AK. *si:ch211-250g4.3*

*si:ch211-250g4.3* encodes a protein with a death like-domain at the N-terminus followed by a FYVE zinc finger domain, a prefoldin domain and three trompomyosin domains. The evolutionary paralog with highest conservation is an uncharacterized gene called CR385063.1 in the Ensembl database. The main vertebrate ortholog associated with *si:ch211-250g4.3* is *ninein*, however zebrafish also contain both a *ninein* gene as well as a *ninein-like* gene that have higher sequences conserved with human and mouse *ninein* than *si:ch211-250g4.3. si:ch211-250g4.3* has not been characterized, but is expressed specifically in the developing vasculature of zebrafish (Figure 3AK’).

AL. *acsbg2*

*acyl-CoA synthetase bubblegum-related 2*, *acsbg2* is an enzyme that preferentially activates oleic and linoleic fatty acids [85]. Although there is a consensus that *acsbg2* is expressed in testis, Zheng et al., 2005b [86] also detected it in human pancreas, liver, small intestine, heart, and kidney, while Fraisl et al., 2006 [87], only detected in testis, and Pei et al., 2006 [85] detected it in testis, and motor neurons in the medulla oblongata and cervical spinal cord. In zebrafish *acsbg2* is expressed in neurons of the spinal cord, as well as the diencephalon, hindbrain and axial vasculature (Figure 3AL’).

AM. *arhgap27*

*arhgap27*, also known as *CIN85 associated multi-domain containing RhoGAP1*, *camgap1*, was initially identified as a binding partner of cin85 by a yeast two hybrid screen, is an active GAP of *cdc42* and *Rac1*, and is implicated in receptor mediated endocytosis [88]. *Arhgap27* is expressed in various tissues in mice including the heart [88], and humans express two splice variants due to alternative splicing [89]. In zebrafish, arhgap27 is vascular specific at 24 hpf (Figure 3AM’).

**References in Supplementary Text**

1. Shimoda Y, Watanabe K (2009) Contactins. Cell Adhesion & Migration 3: 64-70.

2. Kaneko-Goto T, Yoshihara S-I, Miyazaki H, Yoshihara Y (2008) BIG-2 mediates olfactory axon convergence to target glomeruli. Neuron 57: 834-846.

3. Osterfield M, Egelund R, Young LM, Flanagan JG (2008) Interaction of amyloid precursor protein with contactins and NgCAM in the retinotectal system. Development 135: 1189-1199.

4. Zeng L, Zhang C, Xu J, Ye X, Wu Q, et al. (2002) A novel splice variant of the cell adhesion molecule contactin 4 ( CNTN4) is mainly expressed in human brain. J Hum Genet 47: 497-499.

5. Fernandez T, Morgan T, Davis N, Klin A, Morris A, et al. (2004) Disruption of contactin 4 (CNTN4) results in developmental delay and other features of 3p deletion syndrome. Am J Hum Genet 74: 1286-1293.

6. Roohi J, Montagna C, Tegay DH, Palmer LE, DeVincent C, et al. (2009) Disruption of contactin 4 in three subjects with autism spectrum disorder. J Med Genet 46: 176-182.

7. Crozet F, el Amraoui A, Blanchard S, Lenoir M, Ripoll C, et al. (1997) Cloning of the genes encoding two murine and human cochlear unconventional type I myosins. Genomics 40: 332-341.

8. Kim SV, Mehal WZ, Dong X, Heinrich V, Pypaert M, et al. (2006) Modulation of cell adhesion and motility in the immune system by Myo1f. Science 314: 136-139.

9. Zadro C, Alemanno MS, Bellacchio E, Ficarella R, Donaudy F, et al. (2009) Are MYO1C and MYO1F associated with hearing loss? Biochim Biophys Acta 1792: 27-32.

10. Sittaramane V, Chandrasekhar A (2008) Expression of unconventional myosin genes during neuronal development in zebrafish. Gene Expr Patterns 8: 161-170.

11. Yoder JA, Haire RN, Litman GW (1999) Cloning of two zebrafish cDNAs that share domains with the MHC class II-associated invariant chain. Immunogenetics 50: 84-88.

12. Ferro E, Trabalzini L (2010) RalGDS family members couple Ras to Ral signalling and that's not all. Cell Signal 22: 1804-1810.

13. Wong KS, Proulx K, Rost MS, Sumanas S (2009) Identification of vasculature-specific genes by microarray analysis of Etsrp/Etv2 overexpressing zebrafish embryos. Dev Dyn 238: 1836-1850.

14. Isomura M, Okui K, Fujiwara T, Shin S, Nakamura Y (1996) Isolation and mapping of RAB2L, a human cDNA that encodes a protein homologous to RalGDS. Cytogenet Cell Genet 74: 263-265.

15. Peterson SN, Trabalzini L, Brtva TR, Fischer T, Altschuler DL, et al. (1996) Identification of a novel RalGDS-related protein as a candidate effector for Ras and Rap1. J Biol Chem 271: 29903-29908.

16. Post GR, Swiderski C, Waldrop BA, Salty L, Glembotski CC, et al. (2002) Guanine nucleotide exchange factor-like factor (Rlf) induces gene expression and potentiates alpha 1-adrenergic receptor-induced transcriptional responses in neonatal rat ventricular myocytes. J Biol Chem 277: 15286-15292.

17. Ferro E, Magrini D, Guazzi P, Fischer TH, Pistolesi S, et al. (2008) G-protein binding features and regulation of the RalGDS family member, RGL2. Biochem J 415: 145-154.

18. Takaya A, Kamio T, Masuda M, Mochizuki N, Sawa H, et al. (2007) R-Ras regulates exocytosis by Rgl2/Rlf-mediated activation of RalA on endosomes. Mol Biol Cell 18: 1850-1860.

19. Vigil D, Martin TD, Williams F, Yeh JJ, Campbell SL, et al. (2010) Aberrant overexpression of the Rgl2 Ral small GTPase-specific guanine nucleotide exchange factor promotes pancreatic cancer growth through Ral-dependent and Ral-independent mechanisms. J Biol Chem 285: 34729-34740.

20. Lepage SE, Bruce AEE (2008) Characterization and comparative expression of zebrafish calpain system genes during early development. Dev Dyn 237: 819-829.

21. Hata S, Koyama S, Kawahara H, Doi N, Maeda T, et al. (2006) Stomach-specific calpain, nCL-2, localizes in mucus cells and proteolyzes the beta-subunit of coatomer complex, beta-COP. J Biol Chem 281: 11214-11224.

22. Cao Y, Zhao H, Grunz H (2001) XCL-2 is a novel m-type calpain and disrupts morphogenetic movements during embryogenesis in Xenopus laevis. Dev Growth Differ 43: 563-571.

23. Gomez GA, Veldman MB, Zhao Y, Burgess S, Lin S (2009) Discovery and characterization of novel vascular and hematopoietic genes downstream of etsrp in zebrafish. PLoS ONE 4: e4994.

24. Huang S, Gilfillan S, Kim S, Thompson B, Wang X, et al. (2008) MR1 uses an endocytic pathway to activate mucosal-associated invariant T cells. J Exp Med 205: 1201-1211.

25. Huang S, Martin E, Kim S, Yu L, Soudais C, et al. (2009) MR1 antigen presentation to mucosal-associated invariant T cells was highly conserved in evolution. Proc Natl Acad Sci USA 106: 8290-8295.

26. Luo B, Regier DS, Prescott SM, Topham MK (2004) Diacylglycerol kinases. Cell Signal 16: 983-989.

27. Ding L, Traer E, McIntyre TM, Zimmerman GA, Prescott SM (1998) The cloning and characterization of a novel human diacylglycerol kinase, DGKiota. J Biol Chem 273: 32746-32752.

28. Regier DS, Higbee J, Lund KM, Sakane F, Prescott SM, et al. (2005) Diacylglycerol kinase iota regulates Ras guanyl-releasing protein 3 and inhibits Rap1 signaling. Proc Natl Acad Sci USA 102: 7595-7600.

29. Yang Y, Cochran DA, Gargano MD, King I, Samhat NK, et al. (2011) Regulation of flagellar motility by the conserved flagellar protein CG34110/Ccdc135/FAP50. Mol Biol Cell 22: 976-987.

30. Niikura T, Hirata R, Weil SC (1997) A novel interferon-inducible gene expressed during myeloid differentiation. Blood Cells Mol Dis 23: 337-349.

31. Lockyer PJ, Kupzig S, Cullen PJ (2001) CAPRI regulates Ca(2+)-dependent inactivation of the Ras-MAPK pathway. Curr Biol 11: 981-986.

32. Nagase T, Ishikawa K, Miyajima N, Tanaka A, Kotani H, et al. (1998) Prediction of the coding sequences of unidentified human genes. IX. The complete sequences of 100 new cDNA clones from brain which can code for large proteins in vitro. DNA Res 5: 31-39.

33. Zhang J, Guo J, Dzhagalov I, He Y-W (2005) An essential function for the calcium-promoted Ras inactivator in Fcgamma receptor-mediated phagocytosis. Nat Immunol 6: 911-919.

34. Hitomi J, Christofferson DE, Ng A, Yao J, Degterev A, et al. (2008) Identification of a molecular signaling network that regulates a cellular necrotic cell death pathway. Cell 135: 1311-1323.

35. Schultz J, Ponting CP, Hofmann K, Bork P (1997) SAM as a protein interaction domain involved in developmental regulation. Protein Sci 6: 249-253.

36. Hayashi K, Yano H, Hashida T, Takeuchi R, Takeda O, et al. (1992) Genomic structure of the human caldesmon gene. Proc Natl Acad Sci USA 89: 12122-12126.

37. Li Y, Lin JLC, Reiter RS, Daniels K, Soll DR, et al. (2004) Caldesmon mutant defective in Ca(2+)-calmodulin binding interferes with assembly of stress fibers and affects cell morphology, growth and motility. J Cell Sci 117: 3593-3604.

38. Yoshio T, Morita T, Kimura Y, Tsujii M, Hayashi N, et al. (2007) Caldesmon suppresses cancer cell invasion by regulating podosome/invadopodium formation. FEBS Lett 581: 3777-3782.

39. Zheng P-P, van der Weiden M, Kros JM (2005) Differential expression of Hela-type caldesmon in tumour neovascularization: a new marker of angiogenic endothelial cells. J Pathol 205: 408-414.

40. Zheng P-P, Severijnen L-A, Willemsen R, Kros JM (2009) Caldesmon is essential for cardiac morphogenesis and function: in vivo study using a zebrafish model. Biochem Biophys Res Commun 378: 37-40.

41. Kelly MD, Essex DW, Shapiro SS, Meloni FJ, Druck T, et al. (1994) Complementary DNA cloning of the alternatively expressed endothelial cell glycoprotein Ib beta (GPIb beta) and localization of the GPIb beta gene to chromosome 22. J Clin Invest 93: 2417-2424.

42. McKie JM, Sutherland HF, Harvey E, Kim UJ, Scambler PJ (1997) A human gene similar to Drosophila melanogaster peanut maps to the DiGeorge syndrome region of 22q11. Hum Genet 101: 6-12.

43. Zieger B, Hashimoto Y, Ware J (1997) Alternative expression of platelet glycoprotein Ib(beta) mRNA from an adjacent 5' gene with an imperfect polyadenylation signal sequence. J Clin Invest 99: 520-525.

44. Bläser S, Jersch K, Hainmann I, Wunderle D, Zgaga-Griesz A, et al. (2002) Human septin-septin interaction: CDCrel-1 partners with KIAA0202. FEBS Lett 519: 169-172.

45. Caltagarone J, Rhodes J, Honer WG, Bowser R (1998) Localization of a novel septin protein, hCDCrel-1, in neurons of human brain. Neuroreport 9: 2907-2912.

46. Peng X-R, Jia Z, Zhang Y, Ware J, Trimble WS (2002) The septin CDCrel-1 is dispensable for normal development and neurotransmitter release. Mol Cell Biol 22: 378-387.

47. Suzuki G, Harper KM, Hiramoto T, Sawamura T, Lee M, et al. (2009) Sept5 deficiency exerts pleiotropic influence on affective behaviors and cognitive functions in mice. Hum Mol Genet 18: 1652-1660.

48. Bläser S, Röseler S, Rempp H, Bartsch I, Bauer H, et al. (2006) Human endothelial cell septins: SEPT11 is an interaction partner of SEPT5. J Pathol 210: 103-110.

49. Mizutani Y, Kihara A, Igarashi Y (2006) LASS3 (longevity assurance homologue 3) is a mainly testis-specific (dihydro)ceramide synthase with relatively broad substrate specificity. Biochem J 398: 531-538.

50. Reed JC, Doctor KS, Godzik A (2004) The domains of apoptosis: a genomics perspective. Sci STKE 2004: re9.

51. Weber CH, Vincenz C (2001) The death domain superfamily: a tale of two interfaces? Trends Biochem Sci 26: 475-481.

52. Dunlevy JR, Berryhill BL, Vergnes JP, SundarRaj N, Hassell JR (1999) Cloning, chromosomal localization, and characterization of cDNA from a novel gene, SH3BP4, expressed by human corneal fibroblasts. Genomics 62: 519-524.

53. Khanobdee K, Kolberg JB, Dunlevy JR (2004) Nuclear and plasma membrane localization of SH3BP4 in retinal pigment epithelial cells. Mol Vis 10: 933-942.

54. Tosoni D, Puri C, Confalonieri S, Salcini AE, De Camilli P, et al. (2005) TTP specifically regulates the internalization of the transferrin receptor. Cell 123: 875-888.

55. Thalappilly S, Suliman M, Gayet O, Soubeyran P, Hermant A, et al. (2008) Identification of multi-SH3 domain-containing protein interactome in pancreatic cancer: a yeast two-hybrid approach. Proteomics 8: 3071-3081.

56. Bernatchez PN, Acevedo L, Fernandez-Hernando C, Murata T, Chalouni C, et al. (2007) Myoferlin regulates vascular endothelial growth factor receptor-2 stability and function. J Biol Chem 282: 30745-30753.

57. Bernatchez PN, Sharma A, Kodaman P, Sessa WC (2009) Myoferlin is critical for endocytosis in endothelial cells. Am J Physiol, Cell Physiol 297: C484-492.

58. Lu C, Kasik J, Stephan DA, Yang S, Sperling MA, et al. (2001) Grtp1, a novel gene regulated by growth hormone. Endocrinology 142: 4568-4571.

59. Mao M, Biery MC, Kobayashi SV, Ward T, Schimmack G, et al. (2004) T lymphocyte activation gene identification by coregulated expression on DNA microarrays. Genomics 83: 989-999.

60. Bauer H, Willert J, Koschorz B, Herrmann BG (2005) The t complex-encoded GTPase-activating protein Tagap1 acts as a transmission ratio distorter in mice. Nat Genet 37: 969-973.

61. Sommer CA, Pavarino-Bertelli EC, Goloni-Bertollo EM, Henrique-Silva F (2008) Identification of dysregulated genes in lymphocytes from children with Down syndrome. Genome 51: 19-29.

62. Hunt KA, Zhernakova A, Turner G, Heap GAR, Franke L, et al. (2008) Newly identified genetic risk variants for celiac disease related to the immune response. Nat Genet 40: 395-402.

63. Romanos J, Barisani D, Trynka G, Zhernakova A, Bardella MT, et al. (2009) Six new coeliac disease loci replicated in an Italian population confirm association with coeliac disease. J Med Genet 46: 60-63.

64. Smyth DJ, Plagnol V, Walker NM, Cooper JD, Downes K, et al. (2008) Shared and distinct genetic variants in type 1 diabetes and celiac disease. N Engl J Med 359: 2767-2777.

65. Coghill ID, Brown S, Cottle DL, McGrath MJ, Robinson PA, et al. (2003) FHL3 is an actin-binding protein that regulates alpha-actinin-mediated actin bundling: FHL3 localizes to actin stress fibers and enhances cell spreading and stress fiber disassembly. J Biol Chem 278: 24139-24152.

66. Takahashi K, Matsumoto C, Ra C (2005) FHL3 negatively regulates human high-affinity IgE receptor beta-chain gene expression by acting as a transcriptional co-repressor of MZF-1. Biochem J 386: 191-200.

67. Cottle DL, McGrath MJ, Cowling BS, Coghill ID, Brown S, et al. (2007) FHL3 binds MyoD and negatively regulates myotube formation. J Cell Sci 120: 1423-1435.

68. Meeson AP, Shi X, Alexander MS, Williams RS, Allen RE, et al. (2007) Sox15 and Fhl3 transcriptionally coactivate Foxk1 and regulate myogenic progenitor cells. EMBO J 26: 1902-1912.

69. Ding L, Wang Z, Yan J, Yang X, Liu A, et al. (2009) Human four-and-a-half LIM family members suppress tumor cell growth through a TGF-beta-like signaling pathway. J Clin Invest 119: 349-361.

70. Gerber SA, Pober JS (2008) IFN-alpha induces transcription of hypoxia-inducible factor-1alpha to inhibit proliferation of human endothelial cells. J Immunol 181: 1052-1062.

71. Luker KE, Pica CM, Schreiber RD, Piwnica-Worms D (2001) Overexpression of IRF9 confers resistance to antimicrotubule agents in breast cancer cells. Cancer Res 61: 6540-6547.

72. Kraus TA, Lau JF, Parisien J-P, Horvath CM (2003) A hybrid IRF9-STAT2 protein recapitulates interferon-stimulated gene expression and antiviral response. J Biol Chem 278: 13033-13038.

73. Fujii N, Hiraki A, Ikeda K, Ohmura Y, Nozaki I, et al. (2002) Expression of minor histocompatibility antigen, HA-1, in solid tumor cells. Transplantation 73: 1137-1141.

74. Klein CA, Wilke M, Pool J, Vermeulen C, Blokland E, et al. (2002) The hematopoietic system-specific minor histocompatibility antigen HA-1 shows aberrant expression in epithelial cancer cells. J Exp Med 196: 359-368.

75. Spierings E (2008) Minor histocompatibility antigens: targets for tumour therapy and transplant tolerance. Int J Immunogenet 35: 363-366.

76. Bolinger B, Krebs P, Tian Y, Engeler D, Scandella E, et al. (2008) Immunologic ignorance of vascular endothelial cells expressing minor histocompatibility antigen. Blood 111: 4588-4595.

77. Mosavi LK, Cammett TJ, Desrosiers DC, Peng Z-Y (2004) The ankyrin repeat as molecular architecture for protein recognition. Protein Sci 13: 1435-1448.

78. Ma Q, Wang H, Guo R, Wang H, Ge Y, et al. (2006) Molecular cloning and characterization of SRG-L, a novel mouse gene developmentally expressed in spermatogenic cells. Mol Reprod Dev 73: 1075-1083.

79. McClintock TS, Glasser CE, Bose SC, Bergman DA (2008) Tissue expression patterns identify mouse cilia genes. Physiol Genomics 32: 198-206.

80. Ma C, Staudt LM (1996) LAF-4 encodes a lymphoid nuclear protein with transactivation potential that is homologous to AF-4, the gene fused to MLL in t(4;11) leukemias. Blood 87: 734-745.

81. Hiwatari M, Taki T, Taketani T, Taniwaki M, Sugita K, et al. (2003) Fusion of an AF4-related gene, LAF4, to MLL in childhood acute lymphoblastic leukemia with t(2;11)(q11;q23). Oncogene 22: 2851-2855.

82. Wittwer F, van der Straten A, Keleman K, Dickson BJ, Hafen E (2001) Lilliputian: an AF4/FMR2-related protein that controls cell identity and cell growth. Development 128: 791-800.

83. To MD, Faseruk SA, Gokgoz N, Pinnaduwage D, Done SJ, et al. (2005) LAF-4 is aberrantly expressed in human breast cancer. Int J Cancer 115: 568-574.

84. Barton A, Eyre S, Ke X, Hinks A, Bowes J, et al. (2009) Identification of AF4/FMR2 family, member 3 (AFF3) as a novel rheumatoid arthritis susceptibility locus and confirmation of two further pan-autoimmune susceptibility genes. Hum Mol Genet 18: 2518-2522.

85. Pei Z, Jia Z, Watkins PA (2006) The second member of the human and murine bubblegum family is a testis- and brainstem-specific acyl-CoA synthetase. J Biol Chem 281: 6632-6641.

86. Zheng Y, Zhou Z-M, Min X, Li J-M, Sha J-H (2005) Identification and characterization of the BGR-like gene with a potential role in human testicular development/spermatogenesis. Asian J Androl 7: 21-32.

87. Fraisl P, Tanaka H, Forss-Petter S, Lassmann H, Nishimune Y, et al. (2006) A novel mammalian bubblegum-related acyl-CoA synthetase restricted to testes and possibly involved in spermatogenesis. Arch Biochem Biophys 451: 23-33.

88. Sakakibara T, Nemoto Y, Nukiwa T, Takeshima H (2004) Identification and characterization of a novel Rho GTPase activating protein implicated in receptor-mediated endocytosis. FEBS Lett 566: 294-300.

89. Katoh Y, Katoh M (2004) Identification and characterization of ARHGAP27 gene in silico. Int J Mol Med 14: 943-947.
